# Human Immunodeficiency Virus as a Risk Factor for Cardiovascular Disease

**DOI:** 10.1007/s12012-023-09815-4

**Published:** 2023-11-20

**Authors:** Agnieszka Lembas, Andrzej Załęski, Michał Peller, Tomasz Mikuła, Alicja Wiercińska-Drapało

**Affiliations:** 1 Department of Infectious and Tropical Diseases and Hepatology, Medical University of Warsaw, Warsaw, Poland; 2 Hospital for Infectious Diseases, Warsaw, Poland; 3 1st Department of Cardiology, Medical University of Warsaw, Warsaw, Poland

**Keywords:** Antiretroviral Therapy, Human Immunodeficiency Virus, Cardiovascular Disease, Risk Factor

## Abstract

The developments in HIV treatments have increased the life expectancy of people living with HIV (PLWH), a situation that makes cardiovascular disease (CVD) in that population as relevant as ever. PLWH are at increased risk of CVD, and our understanding of the underlying mechanisms is continually increasing. HIV infection is associated with elevated levels of multiple proinflammatory molecules, including IL-6, IL-1β, VCAM-1, ICAM-1, TNF-α, TGF-β, osteopontin, sCD14, hs-CRP, and D-dimer. Other currently examined mechanisms include CD4 + lymphocyte depletion, increased intestinal permeability, microbial translocation, and altered cholesterol metabolism. Antiretroviral therapy (ART) leads to decreases in the concentrations of the majority of proinflammatory molecules, although most remain higher than in the general population. Moreover, adverse effects of ART also play an important role in increased CVD risk, especially in the era of rapid advancement of new therapeutical options. Nevertheless, it is currently believed that HIV plays a more significant role in the development of metabolic syndromes than treatment-associated factors. PLWH being more prone to develop CVD is also due to the higher prevalence of smoking and chronic coinfections with viruses such as HCV and HBV. For these reasons, it is crucial to consider HIV a possible causal factor in CVD occurrence, especially among young patients or individuals without common CVD risk factors.

## Introduction

Human Immunodeficiency Virus (HIV) infection is a chronic disease that is a known risk factor for CVD, a leading cause of mortality worldwide [1]. It is estimated that rates of morbidity and mortality from CVD are 50–100% higher in those with HIV than in a well-matched population without HIV infection [2]. Among the most prevalent cardiovascular conditions in people living with HIV (PLWH) are hypertension, hypercholesterolemia, low HDL-cholesterol, hypertriglyceridemia, and high serum glucose. Moreover, PLWH are more prone to experience ischemic stroke, arrhythmias, heart failure, myocardial infarction, and sudden cardiac death [2, 3]. The mechanisms leading to increased cardiovascular risk in PLWH include viral stimulation of pro-inflammatory molecules, CD4 + lymphocyte depletion, increased intestinal permeability, microbial translocation, and altered cholesterol metabolism [4]. Moreover, the higher prevalence of smoking and other chronic viral coinfections also play important roles in the altered pro-inflammatory status of PLWH [5, 6].

Antiretroviral therapy (ART), the treatment of choice, has significantly contributed to the management of HIV infection and therefore to the increase in life expectancy for PLWH [7]. However, it has been reported that ART can not only suppress the virus and restore immune system function but also may be harmful in terms of cardiovascular risk [8, 9]. Such unfavorable effects depend on the form of ART and the specific drugs applied; however, it is currently believed that antiretroviral therapy plays only a minor role in cardiovascular risk in comparison to HIV itself [9].

Since cardiovascular disease is more prevalent among PLWH than in the general population of the same age, this study aims to explore the finding that early CVD development may be a symptom of HIV infection [10]. In PLWH, similarly to the general population, there are both unmodifiable and modifiable risk factors for CVD. Therefore, HIV infection should be considered in patients with early occurrence of dyslipidemia, hypertension, and high serum glucose levels, with a focus on individuals without the usual risk factors for CVD.

## Unmodifiable CVD Risk Factors in PLWH

### Age

In the general population, age ≥ 45 years for men and ≥ 55 years for women is considered one of the main unmodifiable risk factors of CVD [8]. Studies have reported that multiple chronic comorbidities, including CVD, occur in PLWH approximately a decade earlier than in the general population [10]. There are also reports of increased prevalence of early atherosclerosis and heart failure among PLWH and the beginning of excess heart age in early adulthood [11, 12]. Moreover, studies have suggested that cardiovascular manifestations of HIV infection, especially low HDL cholesterol and hypertriglyceridemia, may already occur in childhood [13]. As a result, the risk of death from CVD appears to be significantly higher among PLWH from 25 to 64 years for every 10-year age group, ranging from a 31% elevated risk among those aged 55–64 years to 202% among those aged 25–34 years in comparison to the general population [14].

### Gender

It has been well documented that in the general population, the risk of CVD is higher in men than in premenopausal women [15]. Interestingly, studies have shown that women living with HIV have 1.5 to 2-fold higher cardiovascular disease risk than men living with HIV [16]. That difference is pronounced in premenopausal women and seems to diminish in old age [17]. The reasons for the increased CVD risk in women seem to be multivariate, and the traditional risk factors may occur more often among women than among men living with HIV. The prevalence of cigarette smoking is higher in women than in men among those living with HIV [18]. Moreover, women living with HIV are more likely to be overweight or obese and to gain weight following antiretroviral therapy compared to men [19, 20]. From a pathophysiological point of view, 59 differentially expressed genes were found in intermediate monocytes in women living with HIV, and these included known atherosclerosis genes such as the liver X receptor gene nuclear receptor subfamily 1 group H member 2 (NR1H2), Nexilin (NEXN), TNF Receptor Associated Factor 1 (TRAF1), Toll-like Receptor 7 (TLR7), and Galectin 3 Binding Protein (LGALS3BP) [21]. Women living with HIV experience menopause earlier than women in the general population, a phenomenon that is associated with an increased risk of visceral fat, reduced muscle mass, and changes in bone density, all of which are HIV-independent but are well-known risk factors for CVD [22].

Studies have shown that transgender women receiving gender-affirming hormonal therapy (GAHT) experience a gain in fat, a decline in lean body mass, and an increase in insulin resistance, all of which are risk factors for cardiovascular disease [23]. In contrast, these effects are not seen in transgender men, possibly because testosterone used as GAHT decreases body fat and increases lean body mass, effects that usually lead to unchanged body mass index (BMI) [24]. Cyproterone acetate, a widely used GAHT in transgender women, leads to an increase in body fat, especially in the android region [24]. Transgender PLWH may be additionally prone to CVD due to other complex factors such as drug-drug interactions with ART, social stressors, and stigma [25–27].

### Race

In the general population, the prevalence of CVD is highest in Black individuals [28]. That association seems to occur also in PLWH. Studies have shown that CVD-related hospitalization rates in PLWH were 45% higher for African Americans than Whites [29]. One of the probable mechanisms explaining this epidemiology is that Black PLWH may be more amenable to inflammation since it has been observed that they experience over 50% higher D-dimer levels while having a detectable HIV viral load in comparison to Whites [30]. Data concerning the mechanism explaining this phenomenon are limited; however, in a study of the general population, several fibrinogen gene polymorphisms, including the Thr312Ala alpha chain variant and the FGG-10,034 C/T variant seem to be associated with 20% higher D-dimer concentrations and may partially explain the racial differences in D-dimer concentration [31].

The lowest hospitalization rates due to CVD have been observed in Asian men living with HIV. Compared to Black men living with HIV, Asians had a three-fold lower rate of hospitalization due to cardiovascular reasons [32]. Those inequalities may result from a lesser number of CVD risk factors among Asians. The cardiovascular health score defined by the American Heart Association comprises seven health factors and behaviors: dietary quality, smoking, physical activity, body mass index, blood pressure, cholesterol, and blood glucose. The average is 0.24 points lower in Asians than Whites and 0.47 points lower than in Blacks [33].

## Modifiable CVD Risk Factors in PLWH

### CD4 + lymphocyte Depletion and Recovery

Untreated HIV infection is associated with a gradual depletion of memory CD4 + lymphocyte count, resulting in higher IL-2 levels and thus increasing the incidence of atherosclerosis and other related inflammatory diseases, including CVD [34]. A lower CD4 + lymphocyte count may be related to the impairment of endothelial function, since it has been reported that circulating microparticles, mostly platelets and endothelial particles, are strongly associated with arterial stiffness in PLWH with advanced immune suppression [35]. A low CD4 + level is also associated with elevated blood pressure, blood glucose, and triglycerides, and decreased HDL cholesterol [36]. There are also reports that PLWH having a CD4 + lymphocyte count < 350 cells/μl have a 30% higher likelihood of having a low HDL cholesterol concentration compared to those with CD4 cell counts > 350 cells/μl [37].

Studies have shown that PLWH with a low CD4 + lymphocyte count have higher proportions of T helper type 17 cells (Th17) and senescent cells, which are associated with higher cardiovascular risk [38]. Senescent cells are known to be connected to atherosclerosis and cardiac fibrosis, and sustained production of Th17 may be a pro-inflammatory factor [39, 40]. The prevalence of clonal hematopoiesis, which has been associated with higher cardiovascular mortality, is higher in PLWH with lower CD4 + lymphocyte counts and residual HIV transcriptional activity [41].

PLWH with lower CD4 + lymphocyte counts has been reported to have low cholesterol efflux and higher sensitivity to C-reactive protein (hs-CRP), both of which are CVD risk factors [42]. Cholesterol efflux capacity is a proinflammatory factor associated with atherosclerosis: a lower cholesterol efflux is negatively associated with the elevation of many proinflammatory molecules, including CRP, fibrinogen, interleukin-6 (IL-6), and serum amyloid A, and positively associated with cardiovascular mortality [43].

Concerning CD4 + lymphocyte count and cardiovascular risk, a special population of patients is those PLWH with poor immunological reconstruction (immunological non-responders; INRs), the patients with persistently lower CD4 + counts and CD4:CD8 ratios despite receiving effective antiretroviral therapy. INRs reportedly have higher rates of mortality due to cardiovascular disease [44]. However, after ART initiation, increases in HDL and LDL-cholesterol levels were observed in INRs, a result that makes it difficult to explicitly assess cardiovascular risk [45].

### Microbial Translocation

Microbial translocation is a hallmark of HIV disease progression. It is defined as the movement of microorganisms or microbial products from the gastrointestinal mucosa into the systemic circulation [46]. The malfunctioning of the barrier leads to an enhanced microbial translocation that further leads to immune activation and inflammation, thereby increasing the risk of cardiovascular disease via pro-inflammatory mechanisms [47].

During infection, the depletion of CD4 + lymphocytes involves the Th17 CD4 + lymphocyte population, the role of which is to defend against various pathogens at mucosal barriers such as the gastrointestinal tract [48]. The depletion of these cells leads to an imbalance of the Th17/Treg ratio and enhanced production of cytokines, including IL-6, IL-17, IL-1β, IL-12, and IL-4, which disrupts epithelial junctions in the gastrointestinal mucosal barrier and therefore leads to increased microbial translocation [49, 50]. There are known markers of microbial translocation, including plasma levels of lipopolysaccharide and soluble CD14, which are bacterial products, and (1➔3)-β-D-Glucan, a fungal product, that are elevated in untreated PLWH [51, 52]. In addition to Th17 CD4 + lymphocytes, the decrease of mucosal-associated invariant T cells (MAIT) induced by chronic inflammation may contribute to increased susceptibility to microbial translocation. It has been suggested that HIV triggers highly activated MAITs to migrate to the colorectal mucosa where they are later subjected to bacteria-induced apoptosis [53]. This phenomenon is followed by increased levels of the proinflammatory cytokines IL-12 and IL-18 and thus an elevated risk of CVD [54].

Other indicators of intestinal damage are intestinal fatty acid-binding protein (I-FABP), zonulin, and regenerating islet-derived protein-3α (REG3α), all of which are considered intestinal permeability markers [55]. Both I-FABP and REG3α plasma levels are significantly elevated in PLWH not receiving ART, and they remain higher even after the introduction of ART compared to healthy controls [56, 57]. REG3α can be used to assess the degree of gut damage and systemic immune activation, and its plasma levels are positively correlated with other proinflammatory biomarkers such as IL-6, IL-8, CXCL13, and IDO-1, the fungal translocation product (1➔3)-β-D-Glucan, and the HIV viral load [58]. Zonulin levels are also elevated in PLWH, causing unclenching of the tight junctions between gut epithelial cells, leading to increases in permeability and macromolecule absorption [59]. I-FABP is involved in the uptake and transport of long-chain fatty acids from the intestinal lumen and may be a marker for mucosal compromise or injury [60]. There are reports that both I-FABP and zonulin can be used to predict mortality in ART-treated PLWH [61].

HIV-related microbial translocation may also be the result of microbial dysbiosis, primarily expressed as decreased diversity or the outgrowth of potentially pathogenic bacteria [62]. A higher proportion of opportunistic pathogens may promote AIDS-related infections, and lower abundances of butyrate-producing bacteria may induce inflammatory bowel disease [63–65]. Likewise, microbial dysbiosis can lead to activation of the gut and peripheral T cells and increases in plasma pro-inflammatory factors such as TNF-α and soluble CD14 [44].

### Dyslipidemia in PLWH

Since the beginning of the HIV epidemic, it has been reported that metabolic syndrome is twice as frequent in PLWH than in the general population [66]. This may be due to altered lipid metabolism causing low HDL cholesterol and hypertriglyceridemia, factors that are considered high-risk lipid profiles for atherosclerosis and cardiovascular disease [67]. Moreover, the prevalence of hypertriglyceridemia, lower HDL cholesterol, and glucose abnormalities are much more common in younger PLWH than in older healthy controls [68]. It has been reported that low HDL cholesterol and hypertriglyceridemia may already occur in children living with HIV [13].

Studies have shown that untreated HIV infection is associated with cardiovascular abnormalities, especially endothelial dysfunction and carotid intima-media thickening [69]. A possible reason for this is the synthesis of Tat by infected cells. The Tat protein elevates the expression levels of IFN-γ, TNF-α, IL-6, and IL-17 and therefore induces apoptosis of endothelial cells. This enables low-density lipoproteins to permeate the sub-endothelial space, thereby causing atherosclerotic lesions [70]. Thus, there is an association between HIV viral load and the risk of dyslipidemia, since the larger number of HIV copies promotes the expression of adhesive proteins and cytokines such as IFN-γ, Il-1β, IL-8, IL-15, and IL-17 [37]. Another possible explanation for the altered lipid metabolism in untreated HIV infection could be the impact of TNF-α decreasing the activity of adipose tissue lipoprotein lipase, an enzyme whose role is to hydrolyze the triacylglycerol component of chylomicrons and VLDL into non-esterified fatty acids and monoacylglycerols [71].

A relationship between lipid metabolism and CD4 + count has also been suggested: PLWH with lower CD4 + lymphocyte counts were reported to have lower concentrations of HDL cholesterol and higher levels of triglycerides than PLWH with higher CD4 + lymphocyte counts [37]. Apart from a low CD4 + lymphocyte count, a history of AIDS-defining events was also reported to be associated with higher total cholesterol and triglyceride concentrations; however, improvement over time has been observed, generally due to the use of lipid-lowering agents [72].

### Hypertension in PLWH

The estimated prevalence of hypertension among PLWH varies from 4 to 50% depending on the country and on the quality of the available data [73]. Despite effective antihypertensive drugs, the achievement of blood pressure control in PLWH remains a challenge [74].

Pathophysiologic mechanisms of hypertension in PLWH are a combination of typical, well-known factors occurring in the general population and the chronic inflammation resulting from HIV infection. It has been reported that higher levels of IL-17 A, IFN-γ, IL-6, and CRP were significantly associated with hypertension in ART-treated PLWH [75]. Moreover, the levels of intermediate monocytes CD14 + 16 + were increased with higher HIV viral load, and this may lead to microbial translocation that drives systemic inflammation [76]. All of these factors enhance the activation of the renin-angiotensin-aldosterone system, a key factor in the development of hypertension [77]. Older age, high BMI, obesity, previous cardiovascular events, chronic kidney disease, a family history of hypertension, and dyslipidemia are traditional risk factors common in PLWH, all of which contribute to the development of hypertension [78].

Besides HIV infection, ART is another risk factor for hypertension in PLWH, since during ART the risk of hypertension is over 1.5-fold higher compared with ART-naïve patients [79]. The negative role of ART in arterial blood pressure involves protease inhibitors (PI) and integrase inhibitors (InSTI) [80]. The use of PIs is associated with carotid artery intima-media thickness and arterial stiffness progression, and InSTIs may promote weight gain and therefore increase the risk of hypertension [81, 82].

### Glucose Metabolism in PLWH

It has been reported that PLWH have higher leptin concentrations, and this may increase central fat mass, worsen insulin sensitivity, and lead to higher glucose levels [83]. Another mechanism of altered glucose metabolism in PLWH may involve lower adiponectin levels that are associated with an increased risk of coronary stenosis [84]. ART may also negatively impact glucose metabolism, as a higher prevalence of insulin resistance was shown in PLWH receiving nucleoside reverse transcriptase inhibitors (NRTI) and PI treatment [85]. PLWH with diabetes mellitus have higher cardiovascular risk according to the Framingham equation and the RAMA-EGAT score, and they more often develop cerebrovascular complications or chronic kidney disease than non-diabetic PLWH [86].

Type 2 diabetes mellitus poses a burden for PLWH, especially for women. It is estimated that the prevalence of diabetes mellitus among women living with HIV is 23% compared with 16% among men living with HIV [87]. Moreover, women living with HIV have a 1.31 greater odds of acquiring diabetes mellitus in comparison to women without HIV infection [87]. Type 2 diabetes, as it involves prolonged hyperglycemia and insulin resistance, impacts the formation of advanced glycation end products and overproduction of reactive oxygen species and activation of protein kinase C, further leading to chronic vascular inflammation resulting in the development of atherosclerotic cardiovascular disease [88].

### Obesity in PLWH and Physical Activity

Obesity as a major metabolic syndrome is one of the traditional risk factors for CVD [89]. A study based on United States registries revealed that among PLWH, 19% of men and 42% of women are obese, while in the general United States population one in three adults is obese [90]. Currently, obesity is less prevalent in PLWH than in the general population; however, the BMI of PLWH has been increasing at a rate more than three times that of the HIV-negative population, and it is estimated that it can soon exceed that of the general population [91].

ART also has a crucial role in increasing the rate of obesity in PLWH [92]. Among widely used antiretroviral agents, weight gain is largely associated with InSTIs, especially bictegravir and dolutegravir [93]. The non-nucleoside reverse transcriptase inhibitors (NNRTIs) such as rilpivirine and NRTIs such as tenofovir alafenamide (TAF) also have a higher potential to cause weight gain than other drugs from these classes of ART [93]. A study analyzing mitochondrial DNA haplogroups in PLWH gaining weight on ART observed that the European haplogroup clade UK and the African haplogroup L3 were associated with significantly greater weight gain after switching to InSTI-based ART [94]. Studies have also suggested the role of direct ART interference with the melanocortin 4 receptor, since modulation of the melanocortin system can influence food intake and body weight. However, these results are currently debatable [95].

Physical activity plays a beneficial role in the reduction of CVD risk in both PLWH and the general population [96]. However, previous observational studies have demonstrated that the level of physical activity of PLWH is lower compared with the general population, a factor that may also have an impact on the increased prevalence of cardiovascular disease [97]. It has been reported that physical activity decreases the risk of CVD and increases the quality of life in PLWH [98].

### Smoking

Smokers are more prone to develop heart failure, atrial fibrillation, venous thromboembolism, and ischemic episodes [6]. The mechanisms by which smoking increases CVD risk generally involve endothelial function, as smoking leads to the impairment of the endothelial cells’ ability to perform repair mechanisms. This in turn results in increased levels of total and apoptotic circulating endothelial microparticles and progenitor cells in smokers [99]. Smoking also impairs endothelium-independent vasodilatation; nitrate-mediated and flow-mediated arterial dilation were lower in smokers than in the non-smoking population [100]. Furthermore, smoking leads to an increased expression of adhesion molecules and proinflammatory cytokines, including IL6, TNF-α, and IL1β [101]. These effects seem to concern not only traditional tobacco smoke; the use of alternative smoking products (e.g., e-cigarettes) was also associated with increased adhesion of monocytes to endothelial cells and increased ICAM-1 and VCAM-1 expression, although with a smaller effect size [102].

Smoking also plays a crucial role in CVD development among PLWH, since PLWH smoke two to three times more than the general population [103]. Smoking is a factor that shortens life expectancy, and in PLWH who smoke, mortality rates of three times those of nonsmokers without HIV infection have been observed. Moreover, tobacco use dramatically increases the mortality risk among PLWH [104]. Additionally, PLWH who smoke tobacco are less likely to quit. One of the possible reasons for the difficulty in quitting is the relatively higher nicotine metabolism in PLWH as measured by the nicotine metabolite ratio (NMR, 3-hydroxycotinine/cotinine). High nicotine metabolism is also responsible for a lower response to transdermal nicotine therapy [105].

### Coinfections

Another important risk factor for CVD development and progression is the presence of coinfections of HIV with other viruses [106]. It has been estimated that approximately 10–15% of the mortalities in PLWH are due to liver diseases, primarily viral hepatitis [107]. Chronic HBV and HCV infections are prevalent among PLWH since the diseases are transmitted through similar routes as HIV [108]. Several reports have suggested that people with HIV/HCV coinfection have elevated levels of plasma inflammation and microbial translocation biomarkers, especially sCD14 and IL-6, compared to PLWH [109]. Patients with HIV/HBV coinfection are more likely to have increased serum TNF-α, IL-6, IL-8, and IL-12p70 concentrations [110]. Additionally, it has been reported that mucosal-associated invariant T cells (MAIT) are depleted in chronic viral infections, a factor that may contribute to increased susceptibility to microbial translocation and therefore to elevated CVD risk [111].

Likewise, the herpesviruses CMV and EBV also appear to play important roles in the risk of CVD in PLWH. CMV can disrupt epithelial junctions in the gastrointestinal tract, thereby enhancing microbial translocation [112]. Chronic CMV infection is also associated with higher serum IL-6 levels and higher proportions of CMV pp65 (NLV)-specific CD8 + T cells [113]. HIV/EBV coinfection was found to be associated with higher IFN-γ, TGF-β1, and IL-2 expression levels [114].

### Metabolic Effects of Antiretroviral Therapy

The most frequently used antiretroviral medication groups are integrase inhibitors, nucleoside reverse transcriptase inhibitors, non-nucleoside reverse transcriptase inhibitors, and protease inhibitors. According to the European AIDS Clinical Society, the majority of currently recommended regimens are based on InSTI. Another first-line treatment is based on doravirine (NNRTI) instead of InSTI. Common alternative regimens allow the usage of darunavir, a protease inhibitor. All recommended schemes of ART contain either one or two NRTIs [115]. Novel ARTs are marked by their high potency, low toxicity, and high effectiveness [116].

Integrase inhibitors are a relatively new class of ART that are supposed to have better efficacy, reduced treatment discontinuation, and higher genetic barrier to drug resistance than older classes of ART [117]. In terms of cardiovascular risk, InSTIs are generally associated with weight gain, obesity, and weight-related comorbidities [118]. Among InSTIs, PLWH taking bictegravir and dolutegravir are at greater risk of weight gain compared to elvitegravir [93]. The demographic factors associated with an increase in BMI are female gender and Black race [93]. Regardless of weight gain, the cardiovascular risk as assessed by the incidence of major adverse cardiac events such as myocardial infarction, ischemic stroke, coronary artery bypass grafting, and percutaneous coronary intervention appears to be decreased among patients receiving InSTI-based regimens in comparison to other classes of ART [119].

The potentially adverse cardiovascular effects of NRTIs are mitophagy-associated endothelial toxicity and mitochondrial oxidative stress [120]. The decrease in mitochondrial DNA copy number in late-passage human aortic endothelial cells and the elevation of senescence-associated β-galactosidase accumulation have been observed in PLWH receiving NRTIs [120]. Moreover, NRTI administration seems to induce increases in the production of reactive oxygen species, accumulation of β-galactosidase, and diminished ATP-linked respiration [121]. The safety profiles of TAF and tenofovir disoproxil (TDF), two widely used forms of tenofovir, show that even a change in the form of the same drug may result in a huge difference in adverse effects. TAF is generally associated with a better safety profile but a possible increase in cardiovascular risk after the switch from ART regimens containing TDF to TAF, especially via increases in total and LDL cholesterol and BMI [93, 122].

Doravirine has beneficial metabolic profiles and can reduce the risk of CVD. Studies have demonstrated decreases in total cholesterol, LDL cholesterol, and triglycerides after switching to doravirine from different regimens [123]. In contrast, PIs are generally considered to have unfavorable effects in terms of cardiovascular risk. The mechanisms responsible include the triggering of reactive oxygen species production, impaired mitochondrial function, and ubiquitin-proteasome system dysregulation, factors that can in turn initiate transcriptional changes that contribute to the perturbation of lipid metabolism [124].

The impact of HIV infection and antiretroviral therapy on chronic inflammation.

#### Proinflammatory Molecules

The molecules associated with HIV that promote inflammation and may lead to immune dysfunction are considered below.

High-sensitivity CRP, one of the most common markers of inflammation, is a well-known risk factor for CVD and a predictor of all-cause mortality [125]. Higher concentrations of hs-CRP in PLWH in comparison to the general population have been demonstrated. Increased levels of D-dimer, a marker of deterioration of CV condition and endothelial dysfunction, are also associated with increased HIV viral load, microbial translocation, immune activation, and mortality risk [126].

Interleukin-6 belongs to the interleukin-6 family, a group of cytokines that includes IL-6, IL-11, IL-27, ciliary neurotrophic factor, leukemia inhibitory factor, oncostatin M, cardiotrophin 1, and cardiotrophin-like cytokine [127]. IL-6 is a pro-inflammatory cytokine in which higher circulating levels are associated with HIV replication [128]. Increased levels of IL-6 are related to the development of CVD and can predict mortality due to CVD or CV events [71]. In HIV infection, IL-1β induces TNF-α and IL-6 expression, leading to sustained proinflammatory responses. HIV is also a factor in the production of IL‐1β via transforming pro-IL-1β into bioactive IL-1β, a cytokine that is associated both with the progression to AIDS and higher CVD risk [129]. A detectable HIV viral load induces a higher TNF-α serum concentration that can initiate and accelerate apoptosis, atherogenesis, thrombosis, vascular remodeling, and oxidative stress and therefore increase cardiovascular risk [130, 131]. TGF-β is related to atherosclerosis-associated vascular inflammation, and the overexpression of TGF-β in PLWH promotes viral replication and plays an important role in the progression of HIV infection and associated diseases [132]. Chronic increase in osteopontin level, reported in PLWH, is another risk factor for CVD, since osteopontin plays a role in the secretion of multiple proinflammatory molecules, including IL-10, IL-12, IL-3, IFN-γ, and can also be used to predict major adverse cardiovascular events [133]. Elevated levels of sCD14 observed in PLWH have been associated with microbial translocation, increased immune activation, and a greater risk of mortality and morbidity due to CVD [134].

The expression of the adhesion molecules VCAM-1 and ICAM-1, which mediate inflammation and promote leukocyte migration, is stimulated by HIV-Tat-1 protein and pro-inflammatory cytokines such as TNF-α and IL-1β [135]. Toll-like receptors activate the expression of VCAM-1 and ICAM-1 in the endothelium, a response that is strongly associated with increased intimal leukocyte accumulation, an important factor in the pathogenesis of human atherosclerosis [136]. VCAM-1 is a diagnostic biomarker of endothelial dysfunction and vascular injury; together with ICAM-1, it has been used in many clinical studies to estimate the risk of CVD [137]. It has been reported that the expression of adhesion molecules in PLWH is significantly higher than in the general population [138].

#### The Impact of Antiretroviral Therapy on Pro-Inflammatory Biomarkers

ART is beneficial in terms of the decrease of the proinflammatory effect induced by HIV infection: studies evaluating the levels of IL-6, IL-1β, D-dimer ICAM-1, VCAM-1, and TNF-α showed a significant decrease in the concentration of those biomarkers in PLWH after receiving antiretroviral therapy. However, the levels of those biomarkers were still elevated in comparison to healthy controls [139–141]. Residual immune activation may continue in compartments such as the central nervous system, the gastrointestinal tract, or the lymph nodes, where ART penetrates insufficiently to completely suppress viral replication, resulting in residual systemic inflammation [142].

**Fig. 1:**
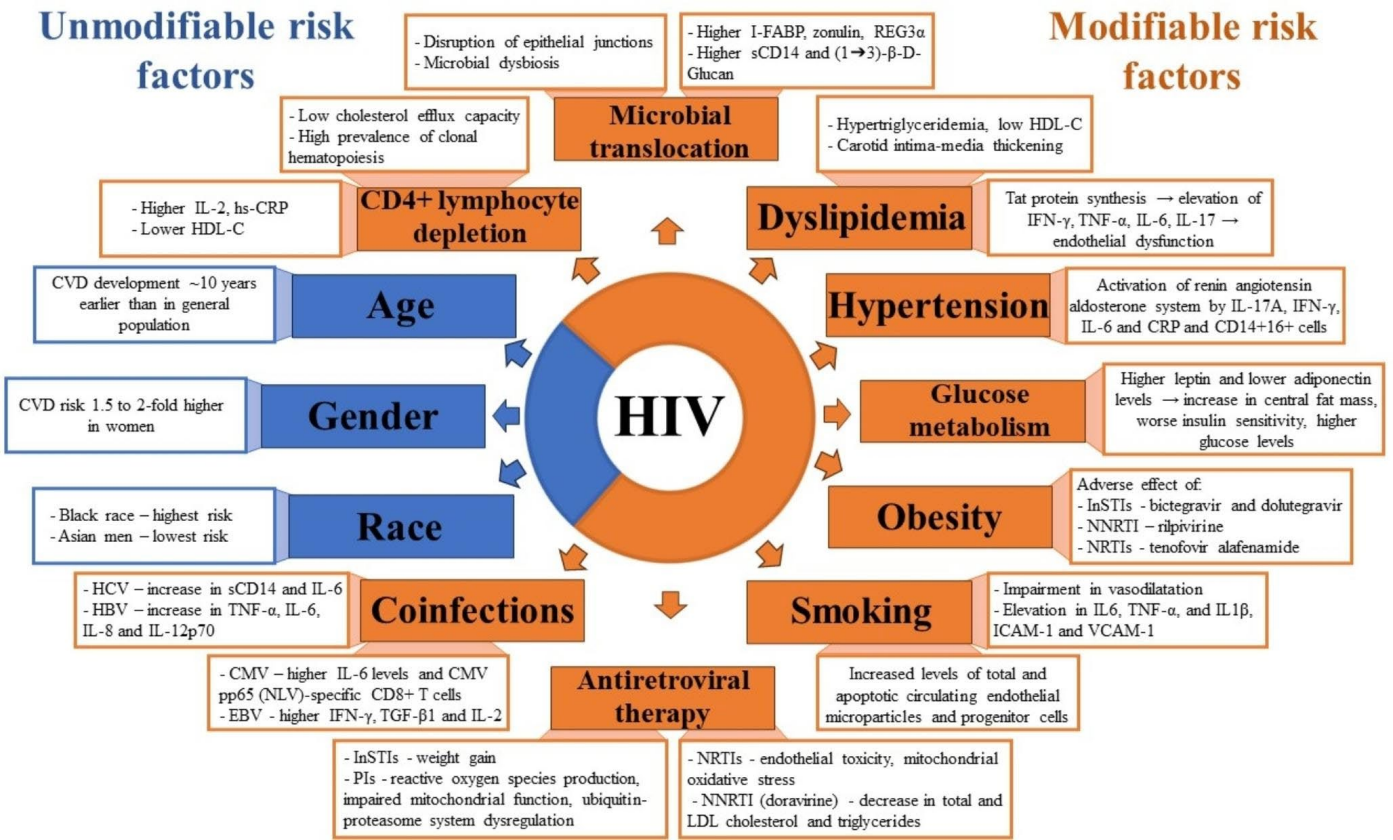
Risk factors for cardiovascular disease among PLWH with underlying mechanisms

The summary of the mechanisms contributing to the elevated risk for cardiovascular disease among PLWH was presented in Fig. 1.

## Conclusions

PLWH experience increased risk of cardiovascular disease, and the reasons for this are multivariate, including the impact of HIV infection itself, the adverse effects of antiretroviral therapy, the ambiguous effect of CD4 + cell count depletion and recovery, and other independent risk factors such as e smoking and chronic viral infections. Although HIV infection is an uncommon disease, clinicians should bear it in mind for people with early occurrence of cardiovascular disease. Dyslipidemia, hypertension, or high serum glucose levels, especially in young patients, should be considered in terms of HIV infection. The cooperation of specialists is crucial for providing the best medical care for people living with HIV and cardiovascular disease.
